# Letter to the Editor From Taylor et al “Type B Insulin Resistance Syndrome: A Rare Cause of Hypoglycemia”

**DOI:** 10.1210/jcemcr/luae001

**Published:** 2024-02-01

**Authors:** Simeon I Taylor, Zhinous Shahidzadeh Yazdi, Elif A Oral

**Affiliations:** Department of Medicine, University of Maryland School of Medicine, Baltimore, MD 21201, USA; Department of Medicine, University of Maryland School of Medicine, Baltimore, MD 21201, USA; Department of Internal Medicine, University of Michigan School of Medicine, Ann Arbor, MI 48105, USA

**Keywords:** autoimmune hypoglycemia, type B insulin resistance syndrome, anti-insulin receptor antibody, C-peptide

Dear Editor,

Bhat et al [1] reported a 59-year-old woman illustrating clinical features of patients with autoantibodies to the insulin receptor (IR-autoantibodies). As described in the first reports of type B insulin resistance [2], the patient exhibited severe insulin resistance and acanthosis nigricans. The patient also exhibited fasting hypoglycemia, which has been reported previously in patients with IR-autoantibodies—either as an isolated symptom [3] or in patients first presenting with insulin-resistant diabetes [4]. In 1982, Taylor et al [3] reported that treatment with high-dose prednisone (120 mg/day) was an effective treatment for hypoglycemia resulting from IR-autoantibodies, with euglycemia being restored within 24 hours. Experiments in an animal model confirmed that glucocorticoid therapy blocked the insulinomimetic activity of the IR-autoantibodies. The beneficial impact of glucocorticoid therapy was confirmed in 2 subsequent case reports [5, 6]. Thus, glucocorticoid therapy might have mitigated the fasting hypoglycemia exhibited by the patient reported by Bhat et al [1].

Kahn et al [7] reported that IR-autoantibodies exhibited insulinomimetic effects in rat adipocytes in vitro. The suggestion that IR-autoantibodies derived from insulin-resistant patients are insulinomimetic seemed surprising and paradoxical when first reported. Subsequently, Taylor and Marcus-Samuels [8] reported that patient-derived IR-autoantibodies decreased the number of insulin receptors on the cell surface—an effect mediated by accelerating the rate of insulin receptor degradation. Because of the existence of “spare receptors,” physiologically relevant activation of insulin receptors is triggered when agonists occupy a small percentage of insulin receptors. Thus, low titers of IR-autoantibodies can trigger hypoglycemia. In contrast, high titers of IR-autoantibodies lead to occupancy of the majority of insulin receptors, which triggers receptor endocytosis and degradation. The associated decrease in the number of insulin receptors at the cell surface leads to extreme insulin resistance. Thus, agonist IR-autoantibodies can trigger severe insulin resistance.

IR-autoantibodies can cause hyperinsulinemia by 2 mechanisms: inhibiting insulin clearance and indirectly promoting insulin secretion. By inhibiting insulin binding to hepatic insulin receptors, IR-autoantibodies inhibit receptor-mediated endocytosis and metabolic clearance of insulin. Thus, because of the inhibition of insulin clearance, patients with hypoglycemia resulting from IR-autoantibodies may have detectable insulin levels even in the face of hypoglycemia-induced suppression of insulin secretion [5, 6]. Because C-peptide is cleared by insulin receptor-independent mechanisms, C-peptide levels are typically low—reflecting suppression of endogenous insulin secretion. In other words, in contrast to what is written in Table 2 in the paper by Bhat et al [1], patients with hypoglycemia resulting from IR-autoantibodies typically manifest laboratory findings at the time of hypoglycemia similar to those of patients with hypoglycemia from injection of exogenous insulin—ie, detectable levels of insulin plus suppressed C-peptide levels. The findings reported by Bhat et al [1] are atypical in that the C-peptide level was not low in their patient. Although the explanation remains unclear, time is required for C-peptide levels to achieve a new steady state in response to hypoglycemia-induced suppression of insulins secretion. Perhaps the patient of Bhat et al [1] had not yet achieved a new steady state reflective of hypoglycemia-induced suppression of insulin secretion.
